# Natively Unstructured Loops Differ from Other Loops

**DOI:** 10.1371/journal.pcbi.0030140

**Published:** 2007-07-20

**Authors:** Avner Schlessinger, Jinfeng Liu, Burkhard Rost

**Affiliations:** 1 Department of Biochemistry and Molecular Biophysics, Columbia University, New York, New York, United States of America; 2 Columbia University Center for Computational Biology and Bioinformatics (C2B2), New York, New York, United States of America; 3 Northeast Structural Genomics Consortium, Columbia University, New York, New York, United States of America; University of California San Diego, United States of America

## Abstract

Natively unstructured or disordered protein regions may increase the functional complexity of an organism; they are particularly abundant in eukaryotes and often evade structure determination. Many computational methods predict unstructured regions by training on outliers in otherwise well-ordered structures. Here, we introduce an approach that uses a neural network in a very different and novel way. We hypothesize that very long contiguous segments with nonregular secondary structure (NORS regions) differ significantly from regular, well-structured loops, and that a method detecting such features could predict natively unstructured regions. Training our new method, NORSnet, on predicted information rather than on experimental data yielded three major advantages: it removed the overlap between testing and training, it systematically covered entire proteomes, and it explicitly focused on one particular aspect of unstructured regions with a simple structural interpretation, namely that they are loops. Our hypothesis was correct: well-structured and unstructured loops differ so substantially that NORSnet succeeded in their distinction. Benchmarks on previously used and new experimental data of unstructured regions revealed that NORSnet performed very well. Although it was not the best single prediction method, NORSnet was sufficiently accurate to flag unstructured regions in proteins that were previously not annotated. In one application, NORSnet revealed previously undetected unstructured regions in putative targets for structural genomics and may thereby contribute to increasing structural coverage of large eukaryotic families. NORSnet found unstructured regions more often in domain boundaries than expected at random. In another application, we estimated that 50%–70% of all worm proteins observed to have more than seven protein–protein interaction partners have unstructured regions. The comparative analysis between NORSnet and DISOPRED2 suggested that long unstructured loops are a major part of unstructured regions in molecular networks.

## Author Summary

The details of protein structures are important for function. Regions that do not adopt any regular structure in isolation (natively unstructured or disordered regions) initially appeared as a curious exception to this structure–function paradigm. It has become increasingly clear that unstructured regions are fundamental to many roles and that they are particularly important for multicellular organisms. Structural biology is just beginning to apprehend the stunning diversity of these roles. Here, we focused on unstructured regions dominated by a particular type of loop, namely the natively unstructured one. We developed a method that succeeded in the distinction between well-structured and natively unstructured loops. For the development, we did not use any experimental data for unstructured regions; when tested on experimental data, the method performed surprisingly well. Due to its different premises, the method captured very different aspects of unstructured regions than other methods that we tested. We applied the new method to two different problems. The first was the identification of proteins that may be difficult targets for structure determination. The second was the identification of worm proteins that have many interaction partners (more than seven) and unstructured regions. Surprisingly, we found unstructured regions of the loopy type in more than 50% of all the promiscuous worm proteins.

## Introduction

### Unstructured Regions Define a New Heterogeneous Structural Reality

**Figure 1:**
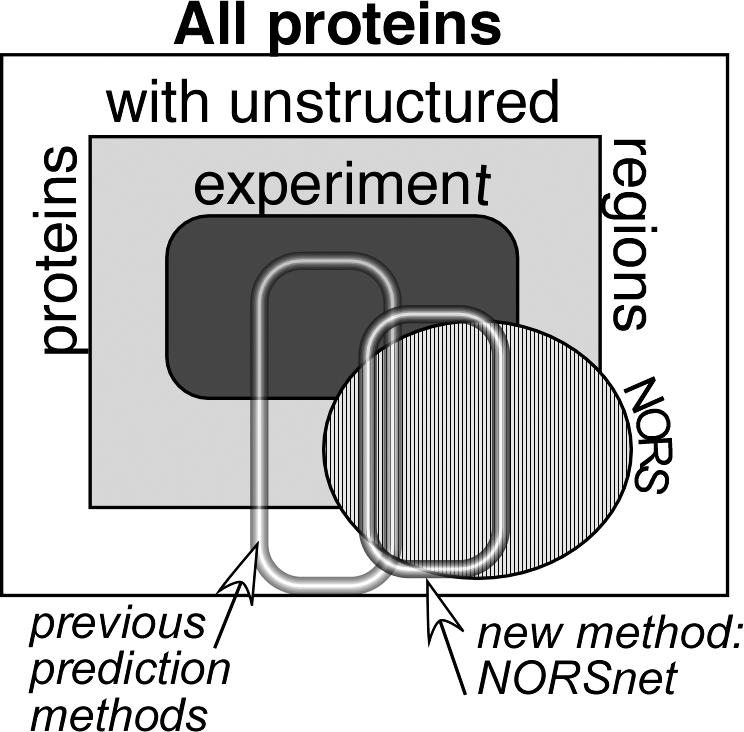
Putative “Map” of Unstructured Regions Proteins with unstructured regions are likely to occupy large portions of sequence space [7,24,27,42] as sketched by the light-gray inner rectangle. The space of all proteins with unstructured regions is likely to be considerably larger than what today's experimental techniques capture. The rounded darker gray rectangle labeled *experiment* sketches proteins for which some experimental method annotated natively unstructured regions. While most NORS regions (predicted long loops, striped gray ellipse) are likely to be natively unstructured, many unstructured regions are not NORS; i.e., they contain helices and strands even in their native form. Previous methods for the prediction of unstructured regions (left lens) are optimized to somehow reflect today's experiments. In contrast, the method introduced here (NORSnet, right lens) is developed based on predictions. This is an advantage because it avoids the bias of today's experimental techniques in a field that is just beginning to grasp its own dimensions, and it is a disadvantage because performance on today's datasets appears somehow limited.

One central paradigm of structural biology is that the intricate details of 3-D protein structures determine protein function [1,2]. In the last few years, many studies have shown that often the lack of a unique, native 3-D structure in physiological conditions can be crucial for function [3–21]. Such proteins are variously called *disordered*, *unfolded*, *natively unstructured*, or *intrinsically unstructured* proteins. A typical example is a protein that adopts a unique 3-D structure only upon binding to an interaction partner and thereby performs its biochemical function [3–6]. The better our experimental and computational means of identifying such proteins, the more we realize that they come in a great variety: some adopt regular secondary structure (helix or strand) upon binding, and some remain loopy. Some proteins are almost entirely unstructured, and others have only short unstructured regions. The more we can recognize short unstructured regions, the more we realize that the term “unstructured protein” would be misleading, as most unstructured proteins have relatively short unstructured regions. There is no single way to define unstructured regions. Here, we define an unstructured region as that which lacks unique 3-D structure by one of the following experimental techniques: circular dichroism (CD) spectroscopy, nuclear magnetic resonance (NMR) spectroscopy, X-ray crystallography, or proteolysis experiments [7–9]. Thanks to the outstanding data collection by the Dunker group, we could also describe this as regions that are the minimal common denominator between all proteins collected in DisProt [10]. However, as we learned from prediction methods, DisProt and similar databases cover only a small fraction of all unstructured regions (Figure 1), and as we learned from recent experiments [11–13], there are many unstructured regions covered neither by these databases nor by existing prediction methods.

### Unstructured Regions Can Be Defined and Recognized in Many Ways

Methods that predict unstructured regions from sequence are mushrooming. Fast methods identify regions with high net charge and low hydrophobicity [14,15], monitor the differences in amino acid propensities between unstructured and other regions (GlobPlot) [16], or identify motifs associated with regions depleted of regular structure [17,18]. Most methods are based on a different definition of disordered region that has been introduced by the Dunker group [19]: residues for which X-ray structures do not have coordinates are considered as disordered. Methods based on this concept used neural networks [19–23] or support vector machines [24]. The meetings for the Critical Assessment of Structure Prediction (CASP) have exclusively assessed disorder predictions on subsets of the “noncoordinate” data [25,26]. The major drawback of this approach is that the Protein Data Bank (PDB) is biased toward proteins for which structures can be determined; natively unstructured proteins are underrepresented in the PDB [5,10,24,27]. This may be one reason why most prediction methods tested by Oldfield et al. [11,12] missed a substantial number of the proteins with unstructured regions identified in a large-scale NMR study spinoff from structural genomics. Other sequence features are predictive of disorder. For example, functionally flexible regions are identified from known structures through molecular dynamics simulation and can be generalized through machine learning. The Wiggle method provides predictions that overlap with unstructured regions even though it is focused on a different aspect of protein flexibility [28].

### Regions with No Regular Secondary Structure Provide Alternative

Our group identified long regions with no regular secondary structure (NORS), which are stretches of 70 or more sequence-consecutive surface residues with few or no predicted helices and strands [27]. NORS regions showed considerable overlap with proteins predicted to have long unstructured regions by various disorder predictors. NORS regions are overrepresented in eukaryotes (over five times more than in prokaryotes), overrepresented in regulatory and interacting proteins [27,29], and share biophysical properties with unstructured regions. In addition, when natively unstructured regions are cocrystallized with their binding partner, they are still enriched in nonregular structure compared with globular proteins; ∼45% and ∼31% of the residues are in coils, respectively [4]. Somewhat surprisingly, the method for predicting regular secondary structure in NORS regions, PROFsec (a profile-based neural network secondary structure predictor) [30–32], accurately predicts the secondary structure state in unstructured regions [4].

NORS regions capture only one particular aspect of unstructured regions (Figure 1). The major advantages of our focus on NORS regions are that this definition implies a simple structural interpretation, and that we can reliably identify thousands of such regions by scanning entire organisms. The thresholds for the minimal length (70 residues) and for the definition of “largely loop” were optimized in order to minimize the identification of any of these stretches in the PDB [27]. This procedure does not explicitly use any information about a protein other than its prediction of secondary structure and solvent accessibility. Thus, it mainly identifies extreme cases (e.g., highly exposed and long loop regions). Since many unstructured regions are shorter, one of our objectives was to capture much shorter NORS-like regions while ascertaining that we would not confuse long, well-structured loops with unstructured regions. One disadvantage of our focus on NORS was that some unstructured regions contain secondary structure elements (helix or strand) [4]; i.e., not all unstructured regions are captured by NORS (Figure 1).

### Eukaryotic Disordered Regions Challenge Structural Genomics

One goal of structural genomics is the determination of a 3-D structure representative for every protein family [33,34]. Unstructured regions have not impeded structural genomics so far because almost all consortia have focused on bacterial proteins in order to increase the structure-to-clone ratio. However, consortia that focus on eukaryotes, such as the Northeast Structural Genomics (NESG) Consortium, or the Center for Eukaryotic Structural Genomics (CESG) have to carefully exclude such problematic targets [35,36]. More than 10,000 proteins have been cloned and more than 3,000 proteins have been purified by NESG. Many of these did not adopt regular structure, possibly because they have unstructured regions that were not filtered out by our original filter, which discarded targets containing NORS regions [29]. To speed up structure determination we need to increase the sensitivity in identifying unstructured regions [11] (i.e., one goal of the development was to end up with a method that would be complementary to existing methods for the identification of unstructured regions).

Our first hypothesis was that NORS regions share commonalities that distinguish such long unstructured loops from well-structured loops. If so, we should be able to somehow distinguish between the two types of loops at least in the sense that all loops predicted to be unstructured by our method ought to have different average features from other loops. We assumed that the neural network would pick up local correlations in amino-acid preferences for the different structural states. Our second hypothesis was that what distinguishes NORS regions from regular loops is exactly what makes regions become unstructured. If so, our method for the identification of NORS regions would also accurately predict unstructured regions.

Here, we describe NORSnet, a new method that extends our NORS concept to also detect shorter (30–70 residues) NORS-like regions. The method was developed without ever using proteins with experimentally known unstructured regions. Instead, it was optimized to distinguish predicted NORS from all other regions. This unique approach, unprecedented in any machine learning method competing in a real-life application with other methods, has three important advantages. First, the data used for development and testing do not overlap. Since NORS regions were predicted from sequence, we can identify thousands of such regions. Our dataset was “dirty” in the sense that it contained many false negatives (all residues in PDB were considered to be well-structured during training) as well as some false positives (incorrect NORS predictions). This was the second major advantage: the positives (unstructured regions) sampled entirely sequenced organisms without any major bias with respect to this particular flavor of unstructured regions. Thereby, we identified unstructured regions that were missed by methods trained on more specialized datasets. The third advantage was that the resulting method explicitly focused on one feature of unstructured regions with a structural interpretation, namely that they are loops. Although we could have assessed NORSnet on any existing dataset due to the lack of overlap, we added a new set with experimental data about unstructured regions different from existing data. Note that both sets differed from each other as well as from the set used for development.

Our three major results confirmed our hypothesis: (1) training on predictions succeeded in developing a powerful prediction method; (2) long loops are a major component of what is picked up by existing methods predicting unstructured regions; and (3) well-ordered and unstructured loops differ. In conjunction with existing methods, the one that we introduce here will allow the focus on particular structural aspects.

## Results/Discussion

### Accurate Distinction between Unstructured and Regular Loop Regions

We trained our system on NORS regions that had been predicted by our previous high-accuracy/low-coverage method [27,29] for the identification of very long regions depleted of predicted helices and strands (NORSp; see Methods). Technically, the task was to separate between all residues predicted to be in a NORS region and all residues in the PDB. As we used neural networks for this task, the typical assessment of accuracy usually involves a cross-validation experiment. For the first time in our work, we did not do this. In fact, we completely ignored the performance of the network on the task it optimized. Our hypothesis simply was that the only aspect that consistently separates extreme NORS regions from all residues in the PDB are the building blocks for a particular type of unstructured regions, namely the NORS-like loopy ones. Therefore, we measured performance on rather different datasets and separation tasks.

First, we established success by predicting *well-structured loops* and *NORS-like loops* for DisProt, which consists of proteins with experimentally characterized unstructured regions. A total of 88% of the residues predicted by NORSnet were also predicted to be loops by PROFsec, while only 51% of the residues predicted as loops in DisProt also appeared NORS-like. In other words, most regions identified by NORSnet appeared to be in loops. Conversely, many loops were not predicted by NORSnet. Since residues in loops were identified through prediction, this difference may have been caused by prediction mistakes. To rule this out, we collected a set of 45 sequence-unique proteins that had been added to the PDB after we had completed developing our method (September 2005 to June 2006). We found that NORSnet classified only 1% of loop residues (Dictionary of Secondary Structure of Proteins states T, S, L) [37] as natively unstructured regions. In other words, NORSnet largely succeeded for these new proteins. In fact, it predicted only one region in these structures to be unstructured, namely a stretch in the HIV type 1 P6 protein of 52 residues [38], the NMR structure of which indicated depletion of regular secondary structure. This protein has been shown to undergo conformational changes [38], suggesting that our method correctly identified it as unstructured.

**Figure 2:**
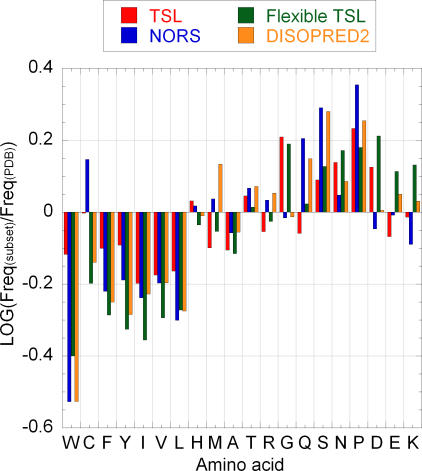
Regular, Flexible, and Predicted-To-Be Unstructured Loops Differed We compared the amino acid compositions between four different subsets representing four types of “loops” (nonhelix/nonstrand): loops from regular, well-ordered structures; i.e., from proteins without natively unstructured regions (states T, S, L from the Dictionary of Secondary Structure of Proteins; in blue); unstructured loops as predicted by NORSnet (in green); “flexible loops” from regular structures (TSL states with normalized B-factors ≥1 [82]; in red); and unstructured regions as predicted by DISOPRED2 (in orange). The sign of the bar corresponds to overrepresentation (positive) or underrepresentation (negative) of amino acids in a subset with respect to the PDB. The NORS and DISOPRED2 residue subsets were taken from the worm genome (from the IntAct database [67]) and were predicted to be unstructured by NORSnet and DISOPRED2. Flexible loops were enriched in amino acids with net charges such as lysine and glutamate (as described before [16,39]). Predicted unstructured regions by NORSnet, however, differed in their composition from regular loops, flexible loops, and from any type of disorder that has been described previously (unpublished data) [39,44]. Cysteines were not overabundant in the unstructured regions predicted by DISOPRED2. Overall, these data suggested that NORSnet captured something other than just “loop” and other than what is captured by methods such as DISOPRED2.

Very long NORS regions differ statistically from regularly structured or well-ordered loops [27]. In general, unstructured regions that are not NORS-like tend to be more loopy than well-structured regions [4]. Here, we showed that our ability to distinguish between well-ordered and unstructured loops was also successful for much shorter loops. Medium-length (30–70 residues) unstructured loops differed from well-structured loops (Figure 2).

NORSnet precisely distinguished between unstructured and well-structured loops. Although the amino acid composition of unstructured loops was similar to that in long disordered regions [39], it was unique (Figure 2). For instance, the regions identified by our method contained significantly more cysteines than other PDB proteins and, within these, more than the set of residues unresolved in electron density maps. Thus, methods trained on unresolved residues, such as DISOPRED2, are likely to miss these regions. Furthermore, methods using pairwise energy potentials, such as IUPred, to predict unstructured regions are also likely to miss these regions, as many cysteines typically coincide with many paired cysteine bonds that significantly contribute to protein stability [40,41].

### Proteins with Unstructured Regions Accurately Identified

About 30%–60% of all eukaryotic proteins have been estimated to contain unstructured regions [24,42]. However, DisProt [10], the largest resource of experimentally verified unstructured regions, contains only a few hundred eukaryotic proteins, and thus covers a small fraction of sequence space (Figure 1). Moreover, this small fraction is not representative, as many unstructured regions described experimentally are missing from existing databases and are not identified by prediction methods [11]. NORSnet attempted to solve both problems by sampling sequence space exhaustively (trained on all positives from entirely sequence organisms) and focusing on unstructured loops.

To assess the accuracy of NORSnet and to estimate to what extent unstructured loops dominate our current identification of unstructured regions, we investigated two different datasets. The first was built around the DisProt database used previously in the literature; the second originated from careful NMR measurements and has not been used in many previous analyses.

#### DisProt dataset.

**Figure 3:**
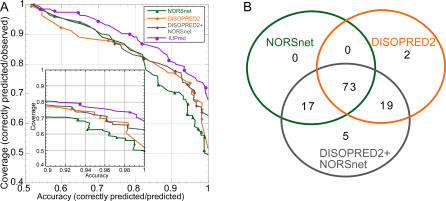
Predictions for DisProt (A) ROC-like curve for NORSnet (green), DISOPRED2 (orange), and their combination (through arithmetic average; gray). While the performance of NORSnet and DISOPRED2 were similar, the combined method seemed to outperform both methods. Particularly, at accuracy = 100% (inset), the combined method covers significantly more sequences than each one of the methods individually. IUPred (purple) outperformed all other methods on this dataset. Note that IUPred was optimized on a set similar to the one used in this study. In contrast, NORSnet and DISOPRED2 were optimized on different sets defining disorder differently. (B) Venn diagram of overlap between very accurate predictions by NORSnet, DISOPRED2, and the combined method. The numbers in the circles are mutually exclusive; for instance, two proteins were identified only by DISOPRED2 to have an unstructured region, and 17 proteins were identified by both NORSnet and by the combined method to have an unstructured region.

The first set included proteins with unstructured regions from DisProt as positives and 173 PDB structures from EVA (a server for assessing protein structure prediction servers) as negatives (see Methods). NORSnet correctly identified half of the DisProt proteins without false positives (Figure 3A). DISOPRED2 [24] was ranked as one of the best three methods for predicting residues that are missing in electron density maps from X-ray crystallography at CASP6 [26] and CASP7 (L. Bordoli, unpublished data). Many other studies corroborated the leading role of DISOPRED2 [22,26,41,43,44]. Overall, NORSnet performed almost on a par with DISOPRED2 for the DisProt dataset (Figure 3A). Simply taking the average over the outputs of DISOPRED2 and NORSnet (DISOPRED2 + NORSnet) outperformed both individually. The improvement was particularly important for the realm of very high accuracy (Figure 3A). IUPred predicts unstructured regions based on a statistical potential optimized for this purpose [41,45]. In our hands, IUPred clearly and consistently outperformed the other methods tested, including the averaged DISOPRED2/NORSnet output (Figure 3A). IUPred is optimized to identify all unstructured regions in DisProt [41,45], but it cannot distinguish between unstructured regions dominated by loops and those dominated by regular secondary structure (as are often found in unstructured regions [4]).

NORSnet predictions were not superior to those from DISOPRED2. However, the performance of these two methods was surprisingly similar despite the fact that NORSnet was not trained on a single experimentally verified unstructured region. Did the similarity in performance indicate that both methods picked up the same signal, i.e., that DISOPRED2 largely captured unstructured loops?

If two prediction methods are based on very different information, their combination typically improves performance over any one of them [44,46]. A more explicit way to demonstrate that methods focus on different aspects is the analysis of their predictions by Venn diagrams. We picked points for which each of the three methods (DISOPRED2, NORSnet, and DISOPRED2 + NORSnet) yielded 100% accuracy and compared the true positives predicted at those thresholds. DISOPRED2 and NORSnet identified the same 73 proteins, but each correctly identified proteins that the other missed (Figure 3B). This agreement supported our initial hypothesis that many unstructured regions are loopy (considerable overlap in true positives). But the most important result was that the two methods complemented each other. At the same 100% accuracy threshold, the combined method (DISOPRED2 + NORSnet) identified more proteins than any of the two individual methods and missed only two proteins that DISOPRED2 correctly identified. Although not surprising given the differences in training set and underlying optimizations, this result highlighted the difference in the types of unstructured regions identified.

**Figure 4:**
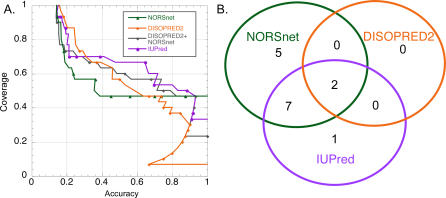
Predictions for NESG Data (A) The NESG set contains many proteins with unstructured regions that are not in DisProt and have never been used for method optimization. We compared NORSnet (in green), DISOPRED2 (in orange), their combined method (in gray), and IUPred (in purple) on these proteins. While DISOPRED2 performed better than all other methods in the low accuracy/high coverage region (top left), the combined method, NORSnet, and IUPred individually excelled in the high accuracy/low coverage region (lower right). (B) Venn diagram of overlap between very accurate predictions by NORSnet, DISOPRED2, and IUPred. The numbers in the circles are mutually exclusive. Note that five proteins were identified only by NORSnet to have an unstructured region.

The combination of DISOPRED2 and NORSnet by averaging their outputs was better than either method alone. This did not work with IUPred and either of the two methods. This might suggest that IUPred covers the same aspects as the other two. However, this notion proved to be incorrect: IUPred missed proteins in the NESG dataset that the others captured (Figure 4B). Therefore, a beneficial combination of different methods predicting unstructured regions may require a more sophisticated algorithm.

#### Unstructured regions from the NESG dataset.

Many prediction methods were optimized or benchmarked on datasets overlapping with DisProt. In contrast, the dataset from the NESG contained proteins with unstructured regions that have not been used for training existing methods yet. The NESG set was collected with a unified definition of unstructured regions based on 2-D NMR experiments [47]; it included 30 proteins with unstructured regions as positives and 170 regular structures solved by NESG as negatives (Methods and Table S1). In the high accuracy region, NORSnet captured a considerable fraction of the positives (40% coverage at 100% accuracy; Figure 4A). The performance of DISOPRED2 was clearly lower than that of NORSnet for high accuracy/low coverage (Figure 4A, lower right), while the inverse was true for low accuracy/high coverage (Figure 4A, upper left). False positives from NORSnet (unstructured regions predicted and not observed) were almost equally divided between X-ray and NMR structures, while DISOPRED2′s false positives were predominantly from NMR structures. The most extreme examples for this were the ordered structures of Methanobacterium thermoautotrophicum 1615 [48] and the conserved domain common to the transcription factors TFIIS, elongin A, and CRSP70 [49].

#### Case study: NORSnet differed from other predictions.

**Figure 5:**
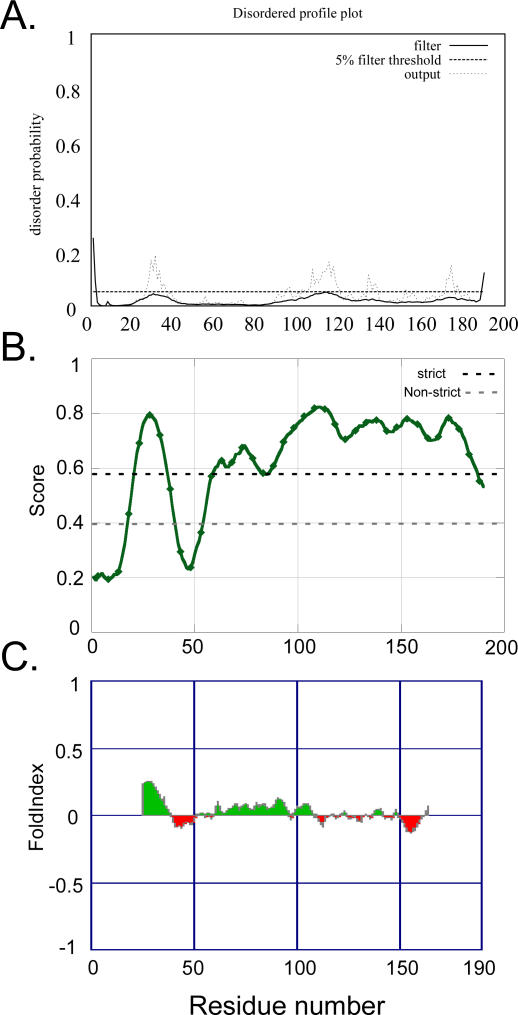
Different Prediction Method Outputs for Kappa-Casein Precursor Kappa-casein precursor has been shown to be unstructured by different experiments [50]. Despite its low content in predicted helices and strands, not all prediction methods identify it as unstructured. We compared outputs of DISOPRED2 (A), NORSnet (B), and FoldIndex (C) for this protein. For DISOPRED2 and NORSnet, higher values indicate unstructured regions; for FoldIndex, low values indicate unstructured regions (red). Note that FoldIndex and DISOPRED2 do not use any explicit information about secondary structure. DISOPRED2 disorder probability, however, is somewhat correlated with coil predictions (Figure S1). DISOPRED2 was not able to distinguish these loops from structured loops. Only NORSnet clearly picked up the strong signal for unstructured regions for most of the protein.

As demonstrated above, NORSnet and other predictors give similar predictions with some exceptions. For instance, we applied NORSnet and two other prediction tools (DISOPRED2 and FoldIndex) on the Kappa-casein precursor protein that is found in milk and stabilizes micelle formation by preventing casein precipitation. Raman optical activity and thermal stability experiments revealed the protein as entirely unstructured in isolation [50]. Secondary structure prediction methods such as PROFsec or PSIPRED [51] predicted the protein to be highly enriched in loops (Figure S1). We may therefore expect that the prediction of the Kappa-casein precursor as unstructured will be a simple task. However, the distinction between natively unstructured and well-structured loops is not trivial: DISOPRED2 did not identify the long *loopy* segment to be part of a natively unstructured region (Figure 5A). In contrast, NORSnet identified most of this protein to be unstructured in its strictest cutoff (corresponding to 100% accuracy on the DisProt dataset; Figure 5B). FoldIndex, a method that uses only amino acid composition and calculates the hydrophobicity/net charge within a given window, predicted only short segments of this protein to be unstructured (Figure 5C).

This example reveals that NORSnet and DISOPRED2 outputs are rather correlated. However, the signal from NORSnet clearly indicated unstructured regions, while the one from DISOPRED2 did not. One reason for this drastic difference may have been that NORSnet correctly captured some global feature from its global input units (see Methods).

#### Natively unstructured loops are elements of domain boundaries.

Although NORSnet was designed to identify all regions in any PDB structure as well-structured, the editor of this manuscript, Phil Bourne, suspected that NORSnet predictions of disorder might more often be in domain boundaries than expected at random and than expected for loop residues in general. To address this, we started with a sequence-unique subset of all PDB proteins considered to be multidomain by SCOP [52] (set taken from [53]). Although a much more comprehensive answer will remain the subject for future investigation, we clearly confirmed this assumption (Figure S4); i.e., the regions in otherwise well-structured proteins that most resemble unstructured regions are domain linkers.

#### Case study: DFF correctly identified despite being a tough case.

**Figure 6:**
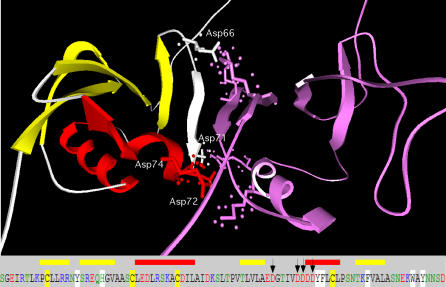
NORSnet Captured Unstructured Regions Related to High Net Charge/Low Hydrophobicity DFF45 (white, yellow, and red) becomes structured upon complex formation with DFF40 (purple; [55]). The interface includes a buried hydrophobic patch surrounded by hydrophilic interactions. Usually, charged residues disrupt the formation of tertiary structure; in this case, however, when the complex is formed, the negative charge of the Asp groups in DFF45 is cancelled out, with the positive charges of DFF40 allowing the protein to be folded. Visualization was done using GRASP2 [85]. Since DFF45 has high secondary structure content, it is a relatively hard target for NORSnet prediction. However, NORSnet correctly identified its unstructured region at a rather stringent cutoff.

The DNA fragmentation factor (DFF) 45 must bind to DFF40 so that DFF40 can execute its catalytic function required for the onset of caspase-mediated apoptosis [54]. The N-terminal domain (NTD) of DFF45 is natively unstructured: its folding is induced upon binding to DFF40 NTD [55] (Figure 6). Methods that only use amino acid composition to predict unstructured regions are likely to perform better on such proteins than more complex prediction methods, since these proteins often have a high net charge which is neutralized upon binding to the target. For example, FoldIndex [15] identified about a third of DFF45 as unstructured.

Secondary structure-prediction methods, such as PSIPRED and PROFsec, usually predict the secondary structure of these regions the way they appear in substrate-bound form. Therefore, methods that use this type of information might be fooled by the rigidity and stability that are associated with regular secondary structure segments and identify these regions as well-structured. Since NORSnet uses secondary structure predictions as input, it may mispredict unstructured regions that become helices and strands upon binding. However, despite the fact that DFF45 NTD is enriched in regular secondary structure (Figure S2), NORSnet identified NTD as an unstructured region at a rather stringent cutoff (the cutoff corresponded to 100% and 97.2% accuracy in the NESG and the DisProt sets, respectively). DISOPRED2 also identified NTD as unstructured, albeit at a less-stringent cutoff (corresponding to 72.2% and 94.2% accuracy).

The unstructured regions in DFF45 are correctly identified by many prediction methods. NORSnet, DISOPRED2, and FoldIndex are only three of those. This example was one of 24 proteins with unstructured regions that become structured upon binding and were extensively analyzed in a recent study [4]. NORSnet identified 14 of these proteins to have unstructured regions in its strictest cutoff. Again, this underlines the surprising finding that methods based on loop predictions can capture unstructured regions of this type. DFF45 and similar proteins are just some of many examples for unstructured regions involved in protein–protein interactions. How representative are they?

### Predicted Unstructured Regions Are Abundant in Protein–Protein Network Hubs

The structural plasticity of proteins with unstructured regions may enable its binding to many proteins, i.e., may typify a protein–protein interaction hub (a protein with many binding partners in an interaction network) [6,56–59]. Several detailed studies have specifically identified unstructured regions in hub proteins that are involved in signaling [3,5,6,60–62]. Natively unstructured regions are also predicted to be abundant in other regulatory processes (e.g., alternative splicing [63] and transcription [64]) and in cancer-associated signaling proteins [65].

**Figure 7:**
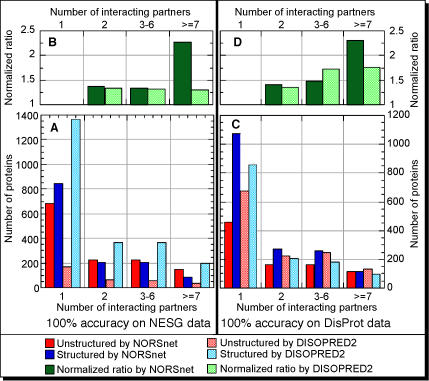
Unstructured Regions Overrepresented in Protein–Protein Hubs of the Worm We ran both NORSnet and DISOPRED2 on worm proteins that are involved in protein–protein interactions (as identified by yeast two-hybrid [66]). The number of proteins that are predicted to be either unstructured or well-structured is plotted against the number of interacting partners for two different thresholds of reliability of the two methods: (A) and (B) were compiled for thresholds at which both methods maintained 100% accuracy for the NESG data (Figure 4), while (C) and (D) were compiled for 100% accuracy on DisProt (Figure 3). Since the number of observed interaction partners falls off dramatically, we had to group the data into bins of roughly equal sizes (*x*-axes). (A) and (C) show the results for the number of proteins predicted in each bin of interaction partners, while (B) and (D) show the normalized ratios to zoom into the difference between unstructured and structured proteins in each bin. These ratios were compiled as Ratio(bin) = {#unstructured(bin) / #structured(bin)} / {#unstructured(1) / #structured(1)}. As all ratios are greater than 1, proteins with more than one interaction partner have more unstructured regions than proteins with one partner. (A) These graphs were compiled with the reliability threshold at which each method achieved 100% accuracy by the NESG data (Figure 4). Overall, this threshold resulted in NORSnet (filled bars) predicting many more proteins with unstructured regions than DISOPRED2 (hatched bars). The difference was particularly relevant for proteins with more interacting partners. (B) NORSnet (filled, dark green) predicted many more unstructured regions in proteins with seven or more interaction partners than did DISOPRED2 (hatched, light green). (C) For the thresholds at which both methods achieved 100% accuracy on the DisProt dataset, DISOPRED2 identified more proteins with unstructured regions than did NORSnet. In contrast to the situation for the NESG set (A), the difference was not as significant for promiscuous proteins (ten or more partners). (D) Although NORSnet (filled, dark green) predicted as many unstructured as structured regions in hubs (seven or more), this ratio was significantly smaller than the one for proteins with a single interaction partner. In other words, even on this dataset NORSnet picked up a much stronger overrepresentation of unstructured regions in hubs than did DISOPRED2 (hatched, light green).

We addressed this point by correlating sustained large-scale datasets of physical protein–protein interactions (see Methods) with predictions for unstructured regions. We applied NORSnet, DISOPRED2, and IUPred to all proteins in the worm *(Caenorhabditis elegans)* proteome and considered only predictions at thresholds corresponding to 100% accuracy. The subset of interacting proteins resulted from the high-throughput experiment by Vidal et al. [66] and from IntAct [67]. Predictions for unstructured regions for all three methods correlated with the average number of interacting partners; in other words, proteins with more unstructured regions had more binding partners (Figure 7). Since we used two different datasets to determine the thresholds for what constituted reliable predictions (DisProt and NESG), we also obtained two different thresholds for each method. For the purpose of fishing for hubs in protein–protein networks, we counted the number of proteins with unstructured regions according to any of those thresholds. Using DisProt to tune thresholds, DISOPRED2 predicted more proteins with unstructured regions than did NORSnet (1279 ± 88 versus 899 ± 76); using the NESG dataset, NORSnet predicted many times more proteins with unstructured regions than did DISOPRED2 (1282 ± 87 versus 321 ± 46; Figure 7). These results agreed with recent studies that estimated hub proteins to be enriched in unstructured regions [57–59]. However, could NORSnet identify any new unstructured regions in hub proteins?

We chose the cutoff that yielded the highest number of unstructured regions (NORSnet, 1,279; DISOPRED2, 1,282) for each method and checked whether the two methods predicted unstructured regions in the same hub proteins. Both methods predicted unstructured regions in most (74) of the proteins observed with more than ten partners (140). DISOPRED2 predicted unstructured regions in another 13 of the promiscuous proteins, and NORSnet in another 21 proteins. If the reliable predictions of both methods are correct, 77% of all promiscuous proteins in the worm (74 + 13 + 21 = 108 of 140) have unstructured regions. While these data do not suffice to identify hubs from sequence, we undoubtedly showed that methods such as NORSnet and DISOPRED2 clearly have some capability in the identification of unstructured regions that will adopt 3-D structures upon binding. While this finding was not new, our particular perspective was that the differences between DISOPRED2 and NORSnet resulted from the difference in the focus of the two. NORSnet focuses more on loopy regions than DISOPRED2, and it also identified more hub proteins. Similar results were obtained when we compared NORSnet and IUPred predictions on the same dataset. Again, IUPred identified the hub signal but much less clearly than did NORSnet (Figure S3). All these observations suggested that the aspect of unstructured regions most relevant to hubs might actually be the unstructured loops.

While NORSnet has some ability to identify unstructured regions that are often involved in binding (Figure 6), it may miss many of these regions due to their enrichment in regular secondary structure (helix, strand) in their bound form. We may therefore wonder why NORSnet identified so many worm hub proteins to have unstructured regions in the first place. Interestingly, many of the hubs had several modules/domains, some of which were predicted not to contain unstructured regions. Some of these modules were DNA-binding domains (such as Homeobox domains) or protein–protein interaction binding motifs (such as EGF repeats). The majority of the unstructured regions predicted by NORSnet in these hubs bridged connections between well-structured domains: these bridges were often on the surface (unpublished data). At first glance, the fact that these regions were predicted to be unstructured might seem biologically unimportant. However, there are several possible biological consequences of the abundance of hubs with unstructured loops. These exposed unstructured/loopy regions might serve as sites for proteolysis, allowing some parts of the protein to undergo proteolytic degradation under different cellular conditions. Such differential degradation could allow different modules of the same protein to be functional under different conditions.

Alternatively, these long connecting loops might function as extremely flexible connecting linkers that facilitate the modules to adopt different orientations, thereby allowing the binding of different targets or binding similar targets in different fashion. Each of these alternatives could be at the heart of a different function. These two hypotheses may explain some of the regulatory characteristics of hub proteins.

#### Mapping the sequence space of proteins with unstructured regions.

Most likely, unstructured regions and NORS regions occupy slightly different parts in sequence space (Figure 1). Indications for the overlap between NORS and unstructured regions are that both are enriched in proline and both depleted of glycine ([39] and Figure 2). Some experimentally observed unstructured regions have been shown to contain cysteines. For instance, Zinc fingers often become structured only upon binding zinc. Nevertheless, most previous studies of unstructured regions did not find cysteines to be overrepresented with respect to well-structured regions in the PDB. This may be due partially to the fact that in well-structured proteins cysteines often stabilize disulfide bonds. Methods optimized to identify regions missing in electron density maps from X-ray crystallography are therefore likely to miss many of the cysteines in unstructured regions. In contrast, NORSnet captured cysteines in unstructured regions (Figure 2). In addition, the differences between DISOPRED2 and NORSnet that were revealed both by our head-to-head comparison on different sets of proteins with unstructured regions and by our analysis of protein hubs pointed to the different types of unstructured regions that we may have to separate (Figure 1). To complicate matters further, some proteins with unstructured regions may look just like any regular protein, while others may be generically different. Consequently, some of the proteins with unstructured regions may be missed by any prediction method.

#### Refining target selection for structural genomics.

One goal of structural genomics projects is to contribute considerably to the increase in the fraction of proteins with known 3-D structures. To achieve this goal, 3-D structures are experimentally determined for representatives of as many large families as possible [33,34,53,68–70]. In particular, the large structural genomics initiatives financed by the Protein Structure Initiative (PSI) from the US National Institutes of Health (NIH) systematically target the experimental determination of structures for large families without representatives of known structure. Structural genomics also aims at making 3-D structures more readily accessible to nonstructural molecular biology and at reducing the costs and difficulty of determining structures. All of these goals require high-throughput determination of 3-D structures. This implies that experimental high-throughput pipelines have to move on if structure determination fails for some families, and that targets are also chosen with the objective to increase the throughput. This does not imply that PSI consortia “go for the low-hanging fruits.” Quite to the contrary, they have succeeded where many small-scale studies have failed.

Membrane proteins and proteins with unstructured regions are the two major types of proteins that are not only avoided by conventional small-scale structural biology but also by structural genomics efforts. Due to the fact that proteins with unstructured regions are much more abundant in eukaryotic organisms, consortia that focus on eukaryotes, such as NESG and CESG, have to carefully avoid such difficult targets. In the last six years, thousands of proteins have been cloned, expressed, and purified by NESG. Although the NESG target selection filtered out many domains with strong predictions for the presence of unstructured regions [35,36], many were left for which biophysical data suggested that they contain unstructured regions [13].

We applied NORSnet to 11,587 putative NESG targets that had already passed our previous and cruder NORS filter (Table 1). Using two different cutoffs, NORSnet predicted that 13%–20% of the previously filtered targets have unstructured regions. Although NORSnet was not optimized to identify very short unstructured regions (≤30 residues), NORSnet predicted 47%–58% of the proteins to contain such regions. The same filter would not have excluded any of the proteins that succeeded in the experimental pipeline, suggesting that the application of NORSnet could have increased the structure–clone ratio. However, the ultimate proof for this assumption will have to wait until another hundred or so experimentally determined structures are added by NESG to the PDB in the next year(s).

**Table 1:**
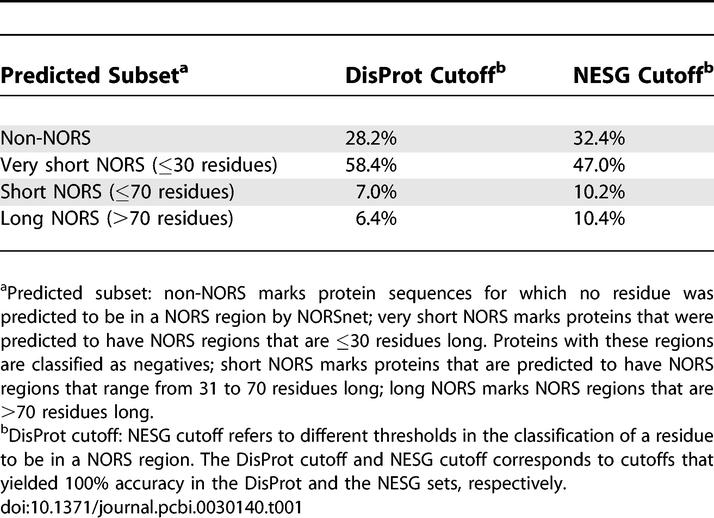
NORSnet Predictions for Structural Genomics Targets

### Conclusions

The intricate details of protein 3-D structures are crucial for their functional role; i.e., structure determines function. Natively unstructured regions do not necessarily contradict this structure–function paradigm. Nevertheless, a variety of proteins require unstructured regions in order to function as domain linkers, filling material, and detergents. For other proteins with unstructured regions, changes in the environment (e.g., pH change, presence of target) or posttranslational modifications can trigger the formation of a regular 3-D structure that will then again determine function. In an evolutionary sense, the required changes/modifications constitute an integral part of the function and are therefore likely to be somehow encoded in the sequence of such proteins. The unusual aspect is that the key structural feature of these proteins is to keep regions natively unstructured or adaptable. The experimental and in silico identification and characterization of proteins with unstructured regions is evolving into an increasingly important challenge for structural biology. In facing this challenge, it becomes increasingly clear that the term “unstructured” describes a rather mixed bag of phenomena from regions that alter between different conformations to those that remain molten globule-like, and from regions that adopt regular helices and strands to those that remain intrinsically loopy.

Here, we present NORSnet, a neural network–based method that revisited the task of identifying unstructured regions from a different angle than that taken by other methods. It focuses on the distinction between unstructured and well-structured loops. The success in this undertaking confirmed our initial hypothesis, namely that short unstructured loops resemble very long unstructured loops (NORS regions). Our application of machine learning was rather unconventional in two ways. First, we trained on positives (predicted NORS) that did not contain the feature we sought to predict (short unstructured loops) and on negatives (all regions in the PDB) that contained regions that we wanted the method to predict as positives; i.e., we implicitly hoped that our development would fail for many cases. Second, we did not optimize any parameters on the dataset used for assessing the performance of our method. Due to the difference in our approach, NORSnet complemented existing methods that optimize on previous datasets of unstructured regions. Consequently, NORSnet will enable the application of additional filters for structural genomics. Last, through a comparison between our new and other prediction methods, we confirmed the importance of unstructured regions for protein–protein interactions. Moreover, we specifically touched on the importance of unstructured loops for network complexity.

## Materials and Methods

### Dataset of NORS regions.

We created our dataset of residues in natively unstructured regions (“positives”) in the following way. We grouped all proteins from 62 entirely sequence proteomes into domain-like families using CHOP and CLUP [35,71,72]. We identified proteins with long NORS regions by the application of NORSp; i.e., all residues that are located in a stretch of >70 consecutive residues with <12% predicted helix or strand [27,29] by PROFsec [30–32] and have at least one contiguous segment longer than ten residues predicted to be on the protein surface [73]. The hope was that all residues in this pool have commonalities that we could extract through machine learning, and that will also be shared by proteins with unstructured regions much shorter than 70 residues. Due to the fact that PROFsec is especially accurate for natively unstructured regions [4], the noise in these data that originated from the prediction mistakes was likely very low. To distinguish between proteins with and without unstructured regions, we needed a set of “negatives” (i.e., residues that are well-structured). For this, we chose a sequence-unique subset of globular protein structures from the PDB. Technically, this sequence-unique subset was taken from the EVA server [74,75]. Specifically, the sequence redundancy was removed above HSSP (a measure for sequence-proximity) similarity values of 0 [76,77] (corresponding to <22% pairwise sequence identity for long alignments). Any pair of sequences between training and testing sets that could be aligned at PSI–BLAST [78] E-values of <10^−3^ according to our standard procedure of three automated iterations [79] was also removed. To further amplify the signal from well-structured regions in the negative set, we also excluded all loops longer than 30 residues. Our datasets were not fully clean in the sense that our negative set of well-structured PDB proteins certainly contained some residues that did not appear in the X-ray structure (which were implicitly treated as well-structured), and that the positive set (predicted NORS) might contain some regular, ordered regions. However, due to the immense size of both datasets and to our use of neural networks, we did not worry about such outliers. In fact, our particular generation of a prediction-based training set that is more than ten times larger, and certainly more representative, than sets used previously might be the most important difference to all previous methods. In the context of a different problem, we showed how beneficial the use of prediction-based sets with errors might be [80].

### Training and testing set.

To optimize the parameters of the method, we trained the network on 90% of the sequences and tested it on the remaining 10%. Note that these data were only used for the development of the method. We never reported the performance of the method on these data. The datasets on which we *did* assess NORSnet had no overlap (HSSP-value <0; i.e., <22% pairwise sequence identity for 250 aligned residues) with any of the proteins used for development. In particular, NORSnet was not optimized in any way on DisProt and the NESG dataset, as these were solely used to assess its performance.

### DisProt data.

After optimizing our method to predict NORS regions (as described below in the prediction method section), we assessed NORSnet performance on different sets without any further optimization. In the first benchmark, we used DisProt proteins that have unstructured regions longer than 30 residues as positives and a sequence-unique subset of 173 PDB X-ray structures as negatives. The latter subset was taken from the EVA server [74,75], and did not include sequences that were in the original training set. One particular advantage of testing our method on DisProt was that we did not have to run any additional cross-validation experiment since we used different proteins; respectively, the same proteins with different labels (*all* residues from PDB in DisProt were explicitly treated as “well-structured” by our training procedure).

### NESG dataset.

To further validate the method, we tested it on a set of proteins from the NESG consortium. The positive set included 30 proteins that were identified to have unstructured regions (“NESG unfolded”), and the negative set included 170 recently determined protein structures. Both sets were identified as such by the NESG consortium. The definition of “unstructured region” was as follows: (1) HSQC (heteronuclear single quantum correlation) was high signal to noise and very low dispersion; and (2) hetNOE (heteronuclear Overhauser effect) data was clean negative (G. T. Montelione, personal communication). Using this set contributed to the removal of two types of biases in DisProt and similar databases. (1) Structure determination method: the negative set was almost equally divided between X-ray and NMR structures. (2) Length bias: while usually sequences selected for NMR structure determination are shorter than for X-ray determination, the NESG consortium reduced this artifact by using both methods in parallel to determine the structures of the same sequences. Thus, the length distribution of the NESG unfolded set is similar to the one of the folded set, in contrast to DisProt database, which consists of some much longer sequences (see Table S1).

### Protein–protein interaction set.

For the large-scale predictions of proteins that are involved in protein–protein interactions, we used the IntAct database (http://www.ebi.ac.uk/intact). IntAct includes both large- and small-scale experiments for different organisms [67]. Specifically, we used proteins from interactions that were detected in a large-scale yeast two-hybrid screen of C. elegans (worm) proteins [66]. The set included 2,622 proteins that participate in 4,039 interactions.

### Prediction method.

We used a standard feed-forward neural network described elsewhere in more detail [30,32,73,81] The crucial novelty for the given task was the choice of input information. This choice was largely influenced by what we found to succeed in different contexts, namely for the prediction of normalized B-values [82] and protein–protein interfaces [83]. Local input information was taken from a sliding window of 13 sequence-consecutive residues (the prediction was for the central residue in that window). For each residue, we used the evolutionary profile (from PSI-BLAST alignments according to our standard protocol [79]), the three-state secondary structure predicted by PROFsec [30–32], the two-state solvent accessibility state predicted by PROFacc (a profile-based neural network predictor of solvent accessibility) [73], and the two-state flexibility prediction by PROFbval [82,84]. Global input information was represented by the global amino acid composition (20 units), the composition in predicted secondary structure (three units), and solvent accessibility (two units), as well the length of the protein/domain-like fragment (three units as in [82]), and the mean hydrophobicity divided by the net charge as was first suggested by Uversky et al. [14].

### DISOPRED2, FoldIndex, and IUPred.

We downloaded the DISOPRED2 package from http://bioinf.cs.ucl.ac.uk/disopred and installed it locally. The package included DISOPRED2 V0.2 and PSIPRED Version 2.45 (from November 2003). To assess its performance on our datasets, we ran the program using the default parameters. The prediction for casein precursor in Figure 5A was taken from the DISOPRED2 server. We ran FoldIndex using the server at http://bip.weizmann.ac.il/fldbin/findex (in September 2006) with default parameters. We ran IUPred using the server at http://iupred.enzim.hu/index.html (in December 2005 and January 2006) with default parameters.

## Supporting Information

Figure S1 PSIPRED Prediction for Kappa-Casein Precursor The protein is predicted to have several long loops (residues 24–42, 89–125, and 130–171). Note that the location of the loops is correlated with high scores predicted by NORSnet and DISORPED2 that use this information.
(7.1 MB TIF)

Figure S2 Secondary Structure Predictions of the N-Termini Domains of DFF45 Despite the fact that the N-term domain of DFF45 is unstructured, PSIPRED predicts secondary structure elements within that region.
(5.0 MB TIF)

Figure S3 Unstructured Regions Overrepresented in Protein–Protein Hubs of Worm Similarly to Figure 7, we ran IUPred on worm proteins that are involved in protein–protein interactions. NORSnet data are identical to those presented in Figure 7. The number of proteins that are predicted to be either unstructured or well-structured is plotted against the number of interacting partners for two different thresholds of reliability of the two methods: (A) and (B) were compiled for thresholds at which both methods maintained 100% accuracy for the NESG data (Figure 4), while graphs (C) and (D) were compiled for 100% accuracy on DisProt (Figure 3). (A) and (C) show the results for the number of proteins predicted in each bin of interaction partners, while (B) and (D) show the normalized ratios to zoom into the difference between unstructured and structured proteins in each bin. These ratios were compiled as Ratio(bin) = {#unstructured(bin)/#structured(bin)} / {#unstructured(1)/#structured(1)}. As all ratios are greater than 1, proteins with more than one interaction partner have more unstructured regions than proteins with one partner. For the thresholds at which both methods achieved 100% accuracy on the DisProt dataset, both IUPred and NORSnet identified unstructured regions in 98 proteins that interact with seven partners or more. IUPred predicted 37 proteins with unstructured regions that NORSnet did not identify, and NORSnet predicted 17 proteins with unstructured regions that IUPred had missed.
(7.0 MB TIF)

Figure S4 NORSnet Captures Domain Boundaries The domain boundaries of 524 multidomain proteins were marked in a procedure described in Liu and Rost [53]. Due to the fact that NORSnet is optimized to identify unstructured stretches that are longer than 30 (and SCOP domain boundaries are often shorter), we used the raw score by NORSnet rather than the filtered output. NORSnet did considerably better than random (in red) and yielded area under the ROC curve (AUC) of 0.672 (in blue). Morever, according to our gold-standard set, termini residues are never defined as domain borders. In “NORSnet no term” (in green), we treated NORSnet outputs of the 60 termini residues in each protein as negatives, assessing only NORSnet predictions for the middle of the chain. The new method was more accurate in distingushing domain boundaries from other residues (AUC = 0.715).
(5.2 MB TIF)

Protocol S1 Synopsis for Supporting Online Material (624 KB DOC)

Table S1 Dataset of Unstructured Proteins from Northeast Structural Genomics Consortium (A) NESG id refers to identifiers given by the NESG consortium.
(B) Disorder signal referred to different levels of signal of a protein to be unstructured from NMR experiments. *Largely* marked largely unstructured proteins; e.g., (1) their HSQC has high signal to noise and very low dispersion and (2) their HetNOE data is clear negative. *Partly* marked partly unstructured proteins, which have some local structure but overall obey the same criteria. A total of 20 proteins were identified as largely unstructured and ten proteins were identified as partly unstructured.
(63.5 KB DOC)

Table S2 PDB Identifiers Used as a Negative Set in Figure 3A (74.5 KB DOC)

### Accession Numbers

The Protein Data Bank (http://www.rcsb.org/pdb) accession numbers for the structures discussed in this paper are HIV type 1 P6 protein (2c55_A), Methanobacterium thermoautotrophicum 1615 (1eij), the conserved domain common to the transcription factors TFIIS, elongin A, and CRSP70 (1eo0), and DFF40 (1ibx).

The DisProt (http://www.disprot.org) accession number for bovine Kappa-casein precursor is DP00192.

## Abbreviations

DFF: DNA fragmentation factor
NMR: nuclear magnetic resonance spectroscopy
NORS: no regular secondary structure
NTD: N-terminal domain
PDB: Protein Data Bank
PSI: Protein Structure Initiative
