# Supplementary material for: Natively Unstructured Loops Differ from Other Loops
Source: PLoS Comput Biol. 2007 Jul 20;3(7):e140. doi: 10.1371/journal.pcbi.0030140 (PMC1924875; doi:10.1371/journal.pcbi.0030140)
Supplement: Supplementary file 1 [file pcbi.0030140.sd001.doc]

Supporting online material

# TOC for Supporting Online Material

TOC for Supporting Online Material	1

Synopsis for Supporting Online Material	1

Figures for Supporting Online Material	3

Fig. S1	3

Fig. S2	4

Fig. S3	5

Fig. S4	6

Tables	7

Table S1	7

Table S2	9

References for Supporting Online Material	10

# Synopsis for Supporting Online Material

Many methods that are optimized to predict natively unstructured regions in proteins are trained and tested on residues that are missing from X-ray structures. It has been shown that residues in these regions are similar in amino acid composition to flexible structured loops (1). Therefore, methods using this approach cannot always distinguish between structured and unstructured loops.

Here, we show one example in which the secondary structure prediction by PSIPRED (2) (Fig. S1) is highly correlated with DISOPRED2 (3) output (Fig. 5A in main text); the locations of the peaks of the prediction are correlated with the locations of the loops. NORSnet, however, is optimized to make the distinction between natively unstructured loops and structured loops (see Fig. 5B).

Furthermore, NORSnet captured the unstructured region in DFF45 in its stringent cutoff despite its enrichment in predicted secondary structure elements (Fig. S2)

Since many disorder predictors are based on different concepts, the predictors often predict different proteins to have unstructured regions (see Fig. 3,4,7). In Fig. S3 we show that both IUPred and NORSnet predict hub proteins to be rich in unstructured regions. Interestingly, each one of the methods reliably predicted different hubs to be unstructured.

# Figures for Supporting Online Material

### Fig. S1

**Fig. S1: PSIPRED prediction for Kappa-casein precursor.** The protein is predicted to have several long loops (residues 24-42, 89-125 and 130-171). Note that the location of the loops is correlated with high scores predicted by NORSnet and DISORPED2 that use this information.

### Fig. S2

**Fig. S2: Secondary structure predictions of the N-termini domains of DFF45.** Despite the fact that the N-term domain of DFF45 is unstructured, PSIPRED predicts secondary structure elements within that region.

### Fig. S3

**Fig. S3: Unstructured regions over-represented in protein-protein hubs of worm.** Similarly to Fig. 7, we ran IUPred on worm proteins that are involved in protein-protein interactions. NORSnet data is identical to the one presented in Fig. 7. The number of proteins that are predicted to be either unstructured or well-structured is plotted against the number of interacting partners for two different thresholds of reliability of the two methods: **A+B** were compiled for thresholds at which both methods maintained 100% accuracy for the NESG data (Fig. 4), while graphs **C+D** were compiled for 100% accuracy on DisProt (Fig. 3). **A+C** show the results for the number of proteins predicted in each bin of interaction partners, while **B+D** show the normalized ratios to zoom into the difference between unstructured and structured proteins in each bin. These ratios were compiled as Ratio(bin)={#unstructured(bin)/#structured(bin)} / {#unstructured(1)/#structured(1)}. As all ratios are above 1, proteins with more than one interaction partners have more unstructured regions than proteins with one partner. For the thresholds at which both methods achieved 100% accuracy on the DisProt dataset, both IUPred and NORSnet identified unstructured regions in 98 proteins that interact with seven partners or more. IUPred predicted 37 proteins with unstructured regions that NORSnet did not identify and NORSnet predicted 17 proteins with unstructured regions that IUPred had missed.

### Fig. S4

**Fig. S4: NORSnet captures domain boundaries.** The domain boundaries of 524 multi-domain proteins were marked in a procedure described in Liu and Rost (4). Due to the fact that NORSnet is optimized to identify unstructured stretches that are longer than 30 (and SCOP domain boundaries are often shorter), we used the raw score by NORSnet rather than the filtered output. NORSnet did considerably better than random (in red) and yielded area under ROC-curve (AUC) 0.672 (in blue). Morever, according to our gold standard set, termini residues are never defined as domain borders. In ‘NORSnet no term’ (in green), we treated NORSnet outputs of the 60 termini residues in each protein as negatives, assessing only NORSnet predictions for the middle of the chain. The new method was more accurate in distingushing domain boundaries from other residues (AUC=0.715).

# Tables

### Table S1

| Number | NESG ID ^a^ | Sequence length | Disorder signal ^b^ |
| --- | --- | --- | --- |
| 1 | AR2242 | 107 | Largely |
| 2 | BhR21 | 117 | Partly |
| 3 | CvR16 | 205 | Partly |
| 4 | FR254 | 163 | Largely |
| 5 | HR1506 | 79 | Largely |
| 6 | HR1538 | 62 | Largely |
| 7 | HR1821 | 157 | Partly |
| 8 | HR1974 | 120 | Largely |
| 9 | HR2078 | 170 | Largely |
| 10 | HR2130 | 173 | Largely |
| 11 | HR224 | 87 | Largely |
| 12 | HR2299 | 113 | Largely |
| 13 | HR36 | 115 | Partly |
| 14 | HR8 | 76 | Largely |
| 15 | HR919 | 208 | Largely |
| 16 | HR922 | 154 | Largely |
| 17 | HR997 | 189 | Largely |
| 18 | KR12 | 231 | Largely |
| 19 | LmR11 | 103 | Partly |
| 20 | MaR51 | 125 | Partly |
| 21 | MhR22 | 75 | Largely |
| 22 | MhR41 | 206 | Partly |
| 23 | MrR47 | 128 | Partly |
| 24 | PsR51 | 76 | Largely |
| 25 | SR128 | 193 | Partly |
| 26 | SmR3 | 62 | Largely |
| 27 | SpR5 | 62 | Largely |
| 28 | WR46 | 193 | Partly |
| 29 | XR5 | 50 | Largely |
| 30 | YR8 | 155 | Largely |

**Table S1: Dataset of unstructured proteins from NorthEast Structural Genomics Consortium**

a	NESG id referred to identifiers given by the NESG consortium.

b	Disorder signal referred to different levels of signal of a protein to be unstructured from NMR experiments. *Largely* marked largely unstructured proteins, e.g., (i) their HSQC has high signal to noise and very low dispersion and (ii) their HetNOE data is clear negative; *partly* marked partly unstructured proteins, which have some local structure but overall obey the same criteria; 20 proteins were identified as largely unstructured and 10 proteins were identified as partly unstructured.

### Table S2

| 1j0w_A | 1uw1_A | 1s5l_I | 1y7y_A | 1r8o_B | 1wpb_A | 1t6a_A | 1zeq_X |
| --- | --- | --- | --- | --- | --- | --- | --- |
| 1nng_A | 1v74_A | 1s5l_J | 1y96_A | 1rfx_A | 1wv8_A | 1t6s_A | 1zhh_B |
| 1nxh_A | 1v74_B | 1s5l_L | 1y9l_A | 1rh5_B | 1wz3_A | 1t71_A | 1zhq_A |
| 1ocs_A | 1vjq_A | 1s5l_M | 1ycy_A | 1rh5_C | 1x0p_A | 1t98_A | 1zlh_B |
| 1ogk_A | 1vk0_A | 1s5l_T | 1yfu_A | 1rhz_A | 1x6i_A | 1t9f_A | 1zoy_D |
| 1oj5_A | 1vk5_A | 1s5l_U | 1ygt_A | 1rk8_C | 1x7v_A | 1tlu_A | 1zpy_A |
| 1ojh_A | 1vrq_D | 1s5l_X | 1yhn_B | 1rli_A | 1x9z_A | 1ttw_A | 1zrl_A |
| 1p57_A | 1w0h_A | 1s5l_Z | 1yle_A | 1rlj_A | 1xg8_A | 1txy_A | 1zv1_A |
| 1pc6_A | 1w2c_A | 1s68_A | 1ylm_A | 1ro5_A | 1xiz_A | 1u14_A | 1zxu_A |
| 1pd3_A | 1w53_A | 1s7b_A | 1yln_A | 1roc_A | 1xk5_A | 1u4h_A | 1zz6_A |
| 1q7l_B | 1w8x_N | 1s7h_A | 1ylq_A | 1rpu_A | 1xl3_A | 1u5k_A | 2a13_A |
| 1q7s_A | 1w8x_P | 1s7i_A | 1ylx_A | 1rr7_A | 1xl3_C | 1u5t_C | 2a1j_A |
| 1q8b_A | 1w94_A | 1sbz_A | 1yn5_A | 1ryl_A | 1xpj_A | 1u7i_A | 2a1x_A |
| 1q8d_A | 1wdu_A | 1sfu_A | 1z0j_B | 1rzn_A | 1xu1_R | 1u84_A | 2a65_A |
| 1q9j_A | 1whz_A | 1sjw_A | 1z0p_A | 1s0y_B | 1xwr_A | 1ud0_A | 2a6q_A |
| 1qw2_A | 1wk2_A | 1sr4_A | 1z1a_A | 1s1h_J | 1xxo_A | 1ufi_A | 2a6q_E |
| 1qz8_A | 1wlf_A | 1sr4_C | 1z21_A | 1s1h_O | 1y0u_A | 1umh_A | 2amy_A |
| 1r0d_A | 1wlq_C | 1ssz_A | 1z2n_X | 1s1i_S | 1y12_A | 1urq_A | 2bem_A |
| 1r2m_A | 1wlz_A | 1swx_A | 1z3i_X | 1s1i_W | 1y5y_A | 1usd_A | 2bho_A |
| 1r4v_A | 1wmi_A | 1sz9_A | 1z67_A | 1s4k_A | 1y66_A | 1ut4_A | 2bjn_A |
| 1r5p_A | 1wmm_A | 1t0f_C | 1zc3_B | 1s5l_H | 1y7m_A | 1utx_A | 2blf_B |
| 1r8g_A | 1wnh_A | 1t0q_C | 1zcd_A | 2bw3_B |  |  |  |

**Table S2: PDB identifiers that were used as negative set in Fig. 3A**

# References for Supporting Online Material

1.	Radivojac, P., Obradovic, Z., Smith, D.K., Zhu, G., Vucetic, S., Brown, C.J., Lawson, J.D. and Dunker, A.K. (2004) Protein flexibility and intrinsic disorder. *Protein Science*, **13**, 71-80.

2.	McGuffin, L.J., Bryson, K. and Jones, D.T. (2000) The PSIPRED protein structure prediction server. *Bioinformatics*, **16**, 404-405.

3.	Ward, J.J., Sodhi, J.S., McGuffin, L.J., Buxton, B.F. and Jones, D.T. (2004) Prediction and functional analysis of native disorder in proteins from the three kingdoms of life. *Journal of Molecular Biology*, **337**, 635-645.

4.	Liu, J. and Rost, B. (2004) Sequence-based prediction of protein domains. *Nucleic Acids Res*, **32**, 3522-3530.
