# Supplementary material for: Natively Unstructured Loops Differ from Other Loops
Source: PLoS Comput Biol. 2007 Jul 20;3(7):e140. doi: 10.1371/journal.pcbi.0030140 (PMC1924875; doi:10.1371/journal.pcbi.0030140)
Supplement: Supplementary file 2 [file pcbi.0030140.st001.doc]

### Table S1

| Number | NESG ID ^a^ | Sequence length | Disorder signal ^b^ |
| --- | --- | --- | --- |
| 1 | AR2242 | 107 | Largely |
| 2 | BhR21 | 117 | Partly |
| 3 | CvR16 | 205 | Partly |
| 4 | FR254 | 163 | Largely |
| 5 | HR1506 | 79 | Largely |
| 6 | HR1538 | 62 | Largely |
| 7 | HR1821 | 157 | Partly |
| 8 | HR1974 | 120 | Largely |
| 9 | HR2078 | 170 | Largely |
| 10 | HR2130 | 173 | Largely |
| 11 | HR224 | 87 | Largely |
| 12 | HR2299 | 113 | Largely |
| 13 | HR36 | 115 | Partly |
| 14 | HR8 | 76 | Largely |
| 15 | HR919 | 208 | Largely |
| 16 | HR922 | 154 | Largely |
| 17 | HR997 | 189 | Largely |
| 18 | KR12 | 231 | Largely |
| 19 | LmR11 | 103 | Partly |
| 20 | MaR51 | 125 | Partly |
| 21 | MhR22 | 75 | Largely |
| 22 | MhR41 | 206 | Partly |
| 23 | MrR47 | 128 | Partly |
| 24 | PsR51 | 76 | Largely |
| 25 | SR128 | 193 | Partly |
| 26 | SmR3 | 62 | Largely |
| 27 | SpR5 | 62 | Largely |
| 28 | WR46 | 193 | Partly |
| 29 | XR5 | 50 | Largely |
| 30 | YR8 | 155 | Largely |

**Table S1: Dataset of unstructured proteins from NorthEast Structural Genomics Consortium**

a	NESG id referred to identifiers given by the NESG consortium.

b	Disorder signal referred to different levels of signal of a protein to be unstructured from NMR experiments. *Largely* marked largely unstructured proteins, e.g., (i) their HSQC has high signal to noise and very low dispersion and (ii) their HetNOE data is clear negative; *partly* marked partly unstructured proteins, which have some local structure but overall obey the same criteria; 20 proteins were identified as largely unstructured and 10 proteins were identified as partly unstructured.
