# Supplementary material for: Natively Unstructured Loops Differ from Other Loops
Source: PLoS Comput Biol. 2007 Jul 20;3(7):e140. doi: 10.1371/journal.pcbi.0030140 (PMC1924875; doi:10.1371/journal.pcbi.0030140)
Supplement: Supplementary file 3 [file pcbi.0030140.st002.doc]

### Table S2

| 1j0w_A | 1uw1_A | 1s5l_I | 1y7y_A | 1r8o_B | 1wpb_A | 1t6a_A | 1zeq_X |
| --- | --- | --- | --- | --- | --- | --- | --- |
| 1nng_A | 1v74_A | 1s5l_J | 1y96_A | 1rfx_A | 1wv8_A | 1t6s_A | 1zhh_B |
| 1nxh_A | 1v74_B | 1s5l_L | 1y9l_A | 1rh5_B | 1wz3_A | 1t71_A | 1zhq_A |
| 1ocs_A | 1vjq_A | 1s5l_M | 1ycy_A | 1rh5_C | 1x0p_A | 1t98_A | 1zlh_B |
| 1ogk_A | 1vk0_A | 1s5l_T | 1yfu_A | 1rhz_A | 1x6i_A | 1t9f_A | 1zoy_D |
| 1oj5_A | 1vk5_A | 1s5l_U | 1ygt_A | 1rk8_C | 1x7v_A | 1tlu_A | 1zpy_A |
| 1ojh_A | 1vrq_D | 1s5l_X | 1yhn_B | 1rli_A | 1x9z_A | 1ttw_A | 1zrl_A |
| 1p57_A | 1w0h_A | 1s5l_Z | 1yle_A | 1rlj_A | 1xg8_A | 1txy_A | 1zv1_A |
| 1pc6_A | 1w2c_A | 1s68_A | 1ylm_A | 1ro5_A | 1xiz_A | 1u14_A | 1zxu_A |
| 1pd3_A | 1w53_A | 1s7b_A | 1yln_A | 1roc_A | 1xk5_A | 1u4h_A | 1zz6_A |
| 1q7l_B | 1w8x_N | 1s7h_A | 1ylq_A | 1rpu_A | 1xl3_A | 1u5k_A | 2a13_A |
| 1q7s_A | 1w8x_P | 1s7i_A | 1ylx_A | 1rr7_A | 1xl3_C | 1u5t_C | 2a1j_A |
| 1q8b_A | 1w94_A | 1sbz_A | 1yn5_A | 1ryl_A | 1xpj_A | 1u7i_A | 2a1x_A |
| 1q8d_A | 1wdu_A | 1sfu_A | 1z0j_B | 1rzn_A | 1xu1_R | 1u84_A | 2a65_A |
| 1q9j_A | 1whz_A | 1sjw_A | 1z0p_A | 1s0y_B | 1xwr_A | 1ud0_A | 2a6q_A |
| 1qw2_A | 1wk2_A | 1sr4_A | 1z1a_A | 1s1h_J | 1xxo_A | 1ufi_A | 2a6q_E |
| 1qz8_A | 1wlf_A | 1sr4_C | 1z21_A | 1s1h_O | 1y0u_A | 1umh_A | 2amy_A |
| 1r0d_A | 1wlq_C | 1ssz_A | 1z2n_X | 1s1i_S | 1y12_A | 1urq_A | 2bem_A |
| 1r2m_A | 1wlz_A | 1swx_A | 1z3i_X | 1s1i_W | 1y5y_A | 1usd_A | 2bho_A |
| 1r4v_A | 1wmi_A | 1sz9_A | 1z67_A | 1s4k_A | 1y66_A | 1ut4_A | 2bjn_A |
| 1r5p_A | 1wmm_A | 1t0f_C | 1zc3_B | 1s5l_H | 1y7m_A | 1utx_A | 2blf_B |
| 1r8g_A | 1wnh_A | 1t0q_C | 1zcd_A | 2bw3_B |  |  |  |

**Table S2: PDB identifiers that were used as negative set in Fig. 3A**
